# Plaster Paris in the Treatment of Fractures

**Published:** 1875-09

**Authors:** E. H. Trickle

**Affiliations:** Syracuse, Ohio


### PLASTER-PARIS IN THE TREATMENT OF FRACTURES.

BY E. H. TRICKLE, M. D., OF SYRACUSE, OHIO.

*Read before the Meigs County (Ohio) Medical Society.*

A short time ago, there was presented to this society a case of *ununited* fracture of the femur, with the following history, so far as relates to the first surgical attention received, and to which the non-union of the bones was attributable:

J. S., æt. 22, in perfect health, and without any bad habits, heriditary taint, or diathesis, whilst at work handling lumber, received a blow from the end of a board, resulting in fracture of the right femur about the junction of the upper and middle third.

He was removed to his home and a neighboring physician summoned, who applied short splints, improvised at the time, and retained them by a roller bandage. A fracture-box was then extemporized, into which the limb was placed—one peculiarity about the box being that it extended from the foot up to, but not beyond the point of fracture. Subsequently, whether from neglect by the medical attendant, or ignorance, on his part, of the requirements of the case, is not known, but the fact remains, the bandages became loosened and the splints misplaced, and, without timely readjustment, a degree of motion was permitted which effectually prevented union of the fractured femur.

The physician, after a few calls and inquiries as to whether the bones had *knit*, abandoned the case to nature, and after a confinement of eleven or twelve weeks, the limb being sometimes in the fracture-box and sometimes out, the splints being sometimes underneath, on top, or all on one side of the limb, the slack of the bandages being occasionally pinned up by the friends, a sort of corset, composed of whalebone stays, stitched into a piece of muslin, was placed around the limb, secured by laces, and the poor unfortunate managed to get around on crutches, dragging a worse than useless limb after him, until he passed into the care of members of the profession more conversant with the principles and improvements of modern surgery, and by whose skill he has been enabled to cast aside his crutches and once more walk the free earth with joyous tread, or trip the light fantastic toe, etc.

The above case is instanced for the reason that it affords a striking illustration of the pernicious results growing out of ignorance, on the part of the surgeon, of the *principles* governing the treatment of such cases, and of the *nature* of the *appliances* necessary to fulfill the indications.

There are *two principles* governing the treatment of fractures, which, if steadily held in view, will generally secure timely and perfect union, no matter how crude or unsightly the means made use of may appear; and should these principles be *ignored*, failure and mortification will as surely be the result, however elegant or popular may be the appliances brought into requisition.

*Apposition* and *immobility* are the principles to be borne in mind, and they are so intimately associated that one cannot be ignored without loosing all the benefits derived from the other. If the surgeon would avoid the "ragged edge" of anxiety, etc., he must not allow the ragged edges of fractured bones to override or depart from each other, but must bring them into *apposition* and retain them immovable until the repairative material is thrown out and consolidated.

With regard to the proper method of securing the *first* of these principles, nothing need be offered, as it is an essential antecedent to the use of *any* of the great variety of appliances made use of in attempting to secure the *second.* But it is proposed to present briefly the merits of an agent which, in the experience of your reporter, has proved most efficient in securing that perfect fixedness of the parts so essential to the welfare and comfort of those undergoing treatment for broken limbs, namely, the use of the plaster-paris bandage or splint.

It is not claimed for this agent that it is a recent discovery in surgery, for it has been before the profession for a number of years, but it can, without doubt, be justly claimed that its merits, as an improvement on other agents, are not sufficiently recognized by a great many of the profession throughout the country. There are several reasons which might be suggested in explanation of this lack of recognition, without detracting from the merits of other agents, a few of which we will enumerate:

1. There are many scientific appliances of real merit in use by many of the profession.

2. Manufacturers and dealers are constantly urging for secular reasons all kinds of inventions, which,

3. Many purchase and exhibit in their offices with pride, and use as occasion requires.

4. Many may have been convinced of its advantages, but never having witnessed its application, shrunk from the attempt, and use what is most familiar to them.

The starch bandage has been fully recognized and appreciated by all who have had extensive experience in surgery, but is often ignored on account of the time required for it to become dry, extension often having to be kept up by assistants for a period of forty to fifty hours.

The *plaster-paris* bandage has all the merits and advantages of the starch bandage, without any of its disadvantages, with the addition of increased porosity and diminished weight.

It is applied very much in the same manner, and sets or hardens in a few minutes, instead of many hours.

When once properly applied in simple fractures, the work of the surgeon has about been finished.

In compound fractures, a section removed, corresponding to the opening of the wound, enables the dressings to be applied and removed with as much facility as though there were no fracture.

It has been the practice of your reporter, when called to a case of fracture, whether simple or compound, and regardless of the stage, to apply this bandage without delay. If the fracture is recent, and there is no extraversation or tumefaction, there will, in all probability, be none worthy of notice. A sheet of cotton wadding, or if this is not at hand any thick, soft cloth, will answer. That part of knit underclothes corresponding to the limb fractured, may be used. The limb being enveloped by one of the above-named agents, taking precaution to avoid wrinkles or creases, and to protect the osseous prominences by additional layers, a roller bandage is next applied, commencing at the distal extremity, and made to include the articulation at the proximal end ^f the fractured bone. When time is not pressing, and assistance ample, the mesher of the roller may be filled with dry plaster-paris, and soaked in water immediately before applying, but this is not essential. The object of the first roller bandage is to give the necessary *support* to the parts, and is all that is required in the way of a *bandage* proper. A quantity of muslin strips of suitable width and length should then be at hand, and then, after smearing the bandage already applied with the plaster-paris, mixed to the consistency of cream, these strips should be smoothly, but rapidly applied, until the necessary strength of the *case* is provided. They should be dipped in the paris mixture as applied, or the mixture applied with the hand, and the strips spread on and moulded to the parts beneath support, or extension is required but a few minutes, for the case hardens very rapidly. In cases where much swelling has taken place, or is apprehended, cotton wadding should, if possible, be procured to envelop the limb, as, from its elastic nature, it accommodates itself to the increase or diminution in size of the limb. Should this not be sufficient, however, the case may be carefully divided, or cut up lengthwise, and a narrow section removed, and then, being somewhat elastic, it may be made to conform to the condition of the limb, whether increasing or diminishing in size.

The advantage of the *starch* bandage over other appliances in the treatment of fractures consists in its conforming accurately to the shape of the limb, and maintaining by its solidity that perfect *immobility* of the parts, set forth as a principle to be observed in the treatment of fractures.

The inconvenience consequent on the length of time necessary for it to become *solid* has often prevented its use, and been followed by the application of other less efficient, but more convenient apparatus, and occasionally with results similar to those described in the case reported in the introduction to this paper.

The substitution of the plaster-paris for the starch bandage remedies all the defects of the latter, and offers decided advantages over it in many other respects.

As we have said, it is *lighter* than the starch bandage.

It is more *porous* in its nature, and therefore more cooling to the enclosed limb. The benefit to the parts, consequent on the evaporation going on during the drying process of the starch bandage, is more than compensated for in the fact that refrigerating lotions may be applied through the ends of or perforations in the plaster-paris bandage from the commencement, and continued indefinitely without affecting its solidity, whilst such applications would soften the starch bandage and render it useless.

It has not been the object of your reporter to detract from the merits of any of the scientific appliances in use by the profession, nor to indulge in adverse criticisms on the variety of special apparatuses *offered* by manufacturers and dealers in such agents, but to urge the more general recognition of what is deemed by him the most efficient, whilst at the same time most inexpensive, means of securing the comfort and welfare of those placed under our care for the treatment of fractures, and also guarding against the possibility of annoyance and chagrin, which so often is the result, when the appliances have been such as do not secure the perfect immobility of the parts, or such as are subject to removal by the caprice of the patient or interference of third parties.

One month ago I was called to attend a child 6 or 7 years old, whose right arm was fractured by a blow from a swing, the humerus being fractured about an inch above the elbow, and a projecting fragment forced through the skin in the flexure of the joint. The fragment was withdrawn, the wound closed with dry cotton, and the fracture reduced by position. A plaster-paris splint was applied, and two days ago I saw the boy in the street playing ball, with his arm bare, the wound having healed by first intention, and the fracture having united kindly.

